# Prognostic Value of MET Gene Copy Number and Protein Expression in Patients with Surgically Resected Non-Small Cell Lung Cancer: A Meta-Analysis of Published Literatures

**DOI:** 10.1371/journal.pone.0099399

**Published:** 2014-06-12

**Authors:** Baoping Guo, Hong Cen, Xiaohong Tan, Wenjian Liu, Qing Ke

**Affiliations:** Department of Chemotherapy, Affiliated Tumor Hospital of Guangxi Medical University, Nanning, People's Republic of China; University of North Carolina School of Medicine, United States of America

## Abstract

**Background:**

The prognostic value of the copy number (GCN) and protein expression of the mesenchymal-epithelial transition (MET) gene for survival of patients with non-small cell lung cancer (NSCLC) remains controversial. This study aims to comprehensively and quantitatively asses the suitability of MET GCN and protein expression to predict patients' survival.

**Methods:**

PubMed, Embase, Web of Science and Google Scholar were searched for articles comparing overall survival in patients with high MET GCN or protein expression with those with low level. Pooled hazard ratio (HR) and 95% confidence intervals (CIs) were calculated using the random and the fixed-effects models. Subgroup and sensitivity analyses were also performed.

**Results:**

Eighteen eligible studies enrolling 5,516 patients were identified. Pooled analyses revealed that high MET GCN or protein expression was associated with poor overall survival (OS) (GCN: HR = 1.90, 95% CI 1.35–2.68, *p*<0.001; protein expression: HR = 1.52, 95% CI 1.08–2.15, *p* = 0.017). In Asian populations (GCN: HR = 2.22, 95% CI 1.46–3.38, *p*<0.001; protein expression: HR = 1.89, 95% CI 1.34–2.68, *p*<0.001), but not in the non-Asian subset. For adenocarcinoma, high MET GCN or protein expression indicated decreased OS (GCN: HR = 1.49, 95% CI 1.05–2.10, *p* = 0.025; protein expression: HR = 1.69, 95% CI 1.31–2.19, *p*<0.001). Results were similar for multivariate analysis (GCN: HR = 1.61, 95% CI 1.15–2.25, *p* = 0.005; protein expression: HR = 2.18, 95% CI 1.60–2.97, *p*<0.001). The results of the sensitivity analysis were not materially altered and did not draw different conclusions.

**Conclusions:**

Increased MET GCN or protein expression was significantly associated with poorer survival in patients with surgically resected NSCLC; this information could potentially further stratify patients in clinical treatment.

## Introduction

Lung cancer continues to be the most common and deadly malignant cancers worldwide [1]. Although important progress in the management of this disease has been observed over the last decade, non-small cell lung cancer (NSCLC) remains a lethal disease, and improving poor prognosis (5-year survival of approximately 15%) remains a challenge [2]. Multiple independent prognostic factors, such as performance status, disease stage, age, sex and amount of weight lost, have previously been identified for predicting survival [3]. Although the use of these factors has been widely accepted, the prognosis of NSCLC is not sufficiently predictable, thus additional prognostic markers are required for more accurate estimation.

The MET gene, located at 7q21-q31, is a potential prognostic genetic marker, which encodes a receptor tyrosine kinase for the HGF/scatter factor (SF) [4]. Met-receptor tyrosine kinase is activated through phosphorylation and the cognate ligand HGF, leading to the activation of a number of downstream pathways, such as the phosphoinositide-3-kinase (PI3K), Ras-Rac/Rho, Ras mitogen-activated protein kinase (MAPK) and phospholipase C-γ signaling pathways, in several types of human cancers, including NSCLC [5]. HGF/Met signaling promotes biological activities, resulting in tumor growth, angiogenesis and the development of invasive phenotypes, making this receptor an attractive target for the potential anti-cancer treatment of NSCLC [6]–[8]. Alterations in the MET gene, including amplification, overexpression and mutations, have been described in a number of solid tumors, including breast and esophageal cancers [9], [10]. The rate of MET amplification in NSCLC remains controversial, ranging from 3% to 10%, depending on the detection method and cut-off criteria [11], [12]. Most studies have indicated a negative prognostic impact of high MET GCN on NSCLC survival [11]–[17], however, other studies have not confirmed this finding [18]-[21]. MET overexpression in NSCLC is variable, ranging from 5% to 75%. Several studies have shown that the overexpression of MET is associated with poor outcome [13], [19], [21]–[26]. However, the prognostic relevance of MET overexpression remains unclear.

With the aim to gain a better insight into the prognostic value of the copy number or protein expression of the MET gene for survival of patients with non-small cell lung cancer, we conducted the first comprehensive meta-analysis of published literature on this topic.

## Materials and Methods

### Identification and selection of relevant studies

PubMed, Embase, Web of Science and Google Scholar were searched for articles concerning the MET GCN, MET protein expression, disease status and survival in patients with NSCLC. The last search update was December 12, 2013. The search strategy included the following medical subject heading terms and keywords variably combined: “Proto-Oncogene Proteins c-met” [Mesh], “Carcinoma, Non-Small-Cell Lung” [Mesh], “MET”, “c-met”, “met Proto-Oncogene Proteins” “Hepatocyte Growth Factor Receptor”, “Scatter Factor Receptor”, “HGF Receptor”, “met gene copy number” “lung cancer”, “NSCLC”, “prognosis”, “prognostic” and “survival”. We did not apply any language restrictions.

Studies meeting the following inclusion criteria were considered for this meta-analysis: (I) Clinical trials and prospective or retrospective cohort studies investigating the correlation of the MET GCN and protein expression status with the OS of NSCLC patients; (II) Measurement methods, including fluorescent *in situ* hybridization (FISH), reverse transcription-polymerase chain reaction (RT-PCR), and immunohistochemistry (IHC); and (III) Findings providing sufficient information for the estimation of hazard ratios and 95% confidence intervals. Only studies published in peer-reviewed journals were included, data from letters and meetings abstracts were not eligible. Two researchers (B.P.G and H.C) independently screened and determined the relevant studies. Any discrepancies were settled through discussion until a consensus was reached.

### Data extraction

Two reviewers independently (B.P.G and H.C) extracted the relevant data from each study and subsequently assessed the data to estimate reliability. The following information was obtained from the MET GCN studies: the first author, year of publication, country of origin, inclusion period, number of patients (Male/Female), age at time of diagnosis (mean, median, range), tumor stage, method of MET GCN detection, cutoff value of high MET GCN, histology, number of patients of high MET GCN, treatment, time of follow-up (median, mean, range), and OS data. The information obtained from each MET protein expression study included the first author, year of publication, country of origin, inclusion period, number of patients (Male/Female), age at time of diagnosis (mean, median, range), tumor stage, method of MET protein expression detection, specimen, cutoff, antibodies, histology, number of patients of high MET protein expression, treatment, time of follow-up (median, mean, and range), and OS data.

### Quality assessment

Two authors (B.P.G and X.H.T) independently assessed the quality of the selected studies using the Newcastle-Ottawa Scale for cohort studies (NOS) [27]. This tool comprises three quality parameters: selection, comparability, and outcome assessment. “Stars” were awarded to demonstrate “high” quality. The stars were subsequently added and used to compare the overall quality in a quantitative manner. A consensus reviewer (H.C) resolved any observed discrepancies.

### Statistical analysis

The primary results were stratified according to MET GCN (high vs. low) and protein expression (high vs. low). The HRs and 95% CIs were combined to obtain the effective value. When the HR was not reported in an article, this parameter was calculated using the methods of Parmar et al [28].

A heterogeneity test based on *I^2^* and Q statistics was performed. The heterogeneity of individual HRs was calculated using *Χ* ^2^ tests according to the method of Peto [29]. Significant heterogeneity was determined at a *p* value less than 0.10. *I^2^* was used to quantify inconsistencies, where a value of 0% indicates no observed heterogeneity, a value less than 25% denotes low heterogeneity, a value from 25.1–50% indicates moderate heterogeneity, and a value greater than 50% indicates substantial heterogeneity [30]. When heterogeneity was observed between primary studies, the random effects model was used. When no heterogeneity was observed, the fixed effects model was used for analysis [31]. HR>1 implies worse survival for the group with high MET GCN or protein expression. The impact of MET on survival was considered statistically significant when the 95% CI did not overlap with 1. Subgroup analyses were performed using different methods to detect the MET GCN and protein expression, conduct univariate and multivariate analyses, and assess the histological subtypes and ethnic source.

Sensitivity analyses were performed to assess the stability of the results. Egger's test [32] was used to detect potential publication bias. Statistical significance was considered for a *p*-value of less than 0.05 for summary HR and publication biases. All calculations were performed using STATA version 11.0 (Stata Corporation, College Station, TX, USA).

## Results

### Eligible studies

**Figure 1:**
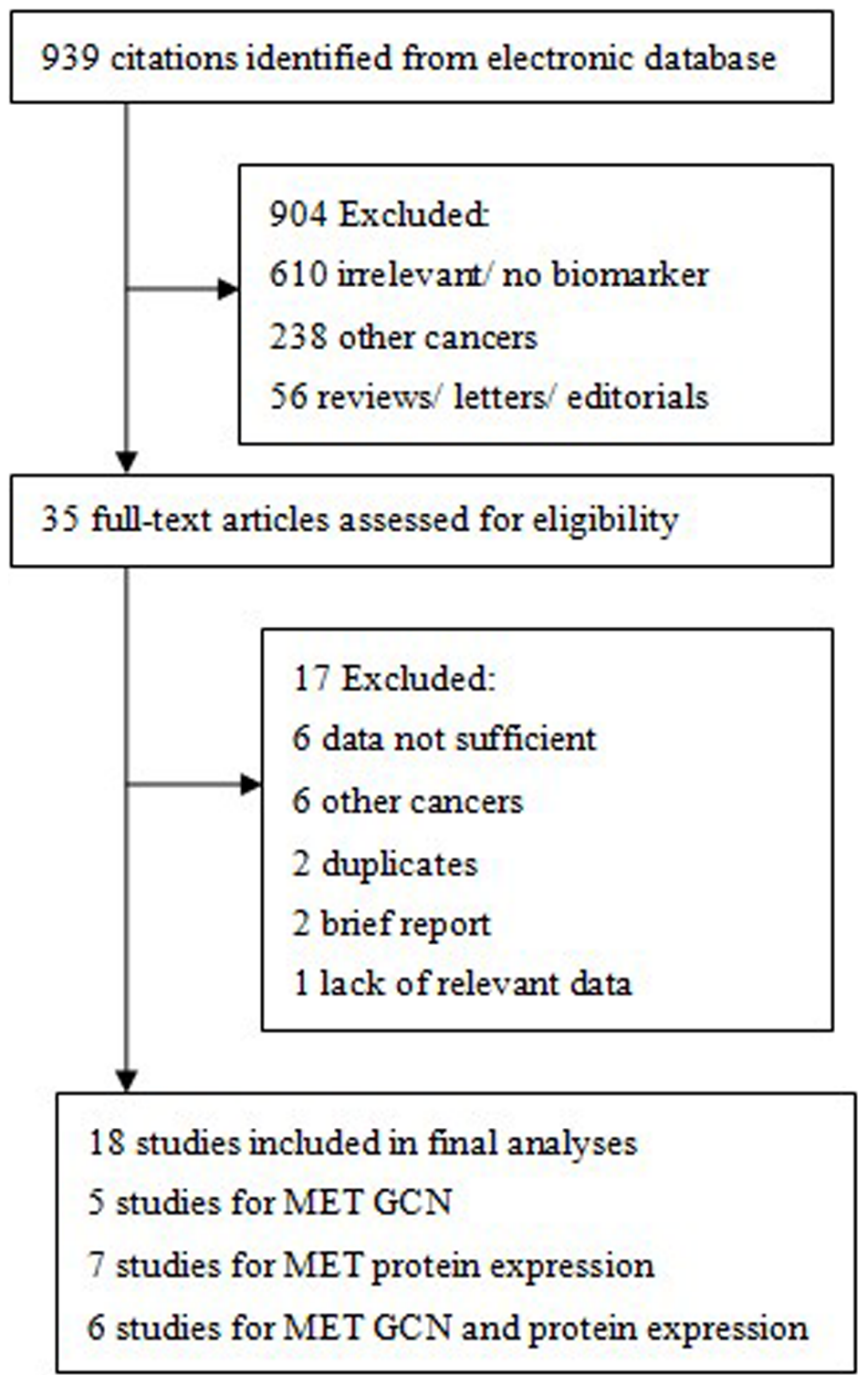
Flow chart of the strategy used for the selection of reports used in our analysis. MET, mesenchymal-epithelial transition; GCN, gene copy number.

A total of 939 records were identified by the primary computerized literature search. After screening the titles and abstracts, thirty-one articles were further reviewed in detail. As indicated in the search flow diagram (Figure 1), 18 studies were finally included in the meta-analysis [11]–[26], [33], [34]. 6 studies provided survival data for both MET GCN and protein expression are listed twice in Table 1 and Table 2 respectively [13], [14], [18]–[20], [21].

**Table 1:** Evaluation of human mesenchymal-epithelial transition (MET) gene copy number in the selected studies.

First author	Year	Country	Inclusion period	No. of Patients (Male/Female)	Age in years	Stage	Method	Cut-off	Histology	No. of patients with high MET	Follow-up (month)	Survival analysis/HR	Adjusted founders
Sun	2013	China	2004–2008	61(47/14)	Mean 58.7 range 32–75	I–IV	RT-PCR	>3 copies	NSCLC	11 (18%)	Mean 29.6±14.7	R (U,M)	TNM stage, MET expression
Dziadziuszko	2012	USA	NA	189(144/45)	Mean 64 range 37–85	I–IV	SISH	Cappuzzo system	NSCLC	14 (10%)	Median 5.3 y; range 1.1–6.9 y	R (U,M)	Demographic and clinical features
Park	2012	Korea	Sep.1994- Dem.2001	380(308/72)	Mean 62 range 54–67	I–IV	FISH	Cappuzzo system or the UCCC criteria	NSCLC	42 (11.1%); 27(7%)	Mean 42.2; range 1–167	R (U,M)	Age, sex, smoking, histology, stage, MET IHC, EGFR IHC, and EGFR FISH
Tanaka	2012	Japan	2004–2009	138(69/69)	NA	I–IV	FISH	Cappuzzo system	ADC	21 (15%); 6 (4%)	≥5 years	E(U)	-
Tsuta	2012	Japan	1997–2007	844(532/312)	Mean 61.7 range 30–88	I–IV	BISH	UCCC criteria	NSCLC	92 (10.9%)	Median 53.3; range 0.3–150	R (U)	-
Tachibana	2012	Japan	2001–2008	106(51/55)	Median 64 range 31–89	I–III	FISH	>3 copies	ADC	11(10.4%)	Median 50; range 5–99	R (U,M)	Pleural or vascular invasion, lymphatic permeation, nuclear grading, immunoreactivity for MET and HGF
Chen	2011	Taiwan	Jan.1996- May 2004	208(128/80)	Median 65	I–IV	FISH	MET gene copies ≥3	NSCLC	22 (10.58%)	Range 27days-158 months	R (U,M)	Age, sex, smoking, histology, stage, EGFR copy, EGFR and KRAS mutations
Onitsuka	2010	Japan	2003–2007	183(102/81)	Mean 68.5 range 23–88	IA–IIIB	RT-PCR	≥1.31 copies	ADC	8 (4%)	Median 34.2; range 1.0–70.0	R (U,M)	Sex, age, stage, EGFR and KRAS mutations, p-MET, HGF expression
Go	2010	Korea	Jan.1995- Jan.2000	97(90/7)	Median 60 range 28–81	I–IV	FISH	Cappuzzo system or the UCCC criteria	SCC	12 (6.7%)	Median 56; range 1–121	R (U,M)	Age, sex, smoking, stage, EGFR FISH
Cappuzzo	2009	Italy	2000–2004	447(373/74)	Median 66 range 33–86	I–IV	FISH	Cappuzzo system	NSCLC	48 (11.1%)	Median 43.9	R (U,M)	Sex, smoking, histology, stage, grade, EGFR FISH
Okuda	2008	Japan	1997–2007	213(148/65)	Median 66 range 33–88	I–IV	RT-PCR	>3 copies	NSCLC	12 (5.6%)	≥5 years	E (U), R(M)	Age, sex, smoking, histology, stage, differentiation, EGFR mutations

NA: not available; NSCLC, non-small cell lung cancer; ADC, adenocarcinoma; SCC, squamous cell carcinoma; RT-PCR, real-time polymerase chain reaction; FISH, fluorescent in situ hybridization; SISH, silver in situ hybridization; BISH, bright-field in situ hybridization; IHC, immunohistochemistry; Cappuzzo scoring system: MET FISH-positive group was defined mean MET gene copy number≥5 copies per cell; UCCC criteria: the University of Colorado Cancer Center) criteria, MET gene status was classified into two groups according to the frequency of tumor cells with specific copy numbers of the MET gene and the chromosome 7 centromere: FISH-positive MET MET to CEP7 ratio ≥2; >15 copies of the MET signals in >10% of tumor cells; small gene cluster [4–10 copies]; or innumerable tight gene clusters in >10% the tumor cells); EGFR, epidermal growth factor receptor; HR: hazard ratio, obtained by estimated (E) or reported in text (R). “M” means the HR come from multivariate analysis, and “U” means HR come from univariate analysis.

**Table 2:** Evaluation of human mesenchymal-epithelial transition (MET) by immunohistochemistry (IHC) in the selected studies in the selected studies.

First author	Year	Country	Inclusion period	No. of Patients(Male/Female)	Age in years	Histology	Stage	Method	Specimen	Cut-off	Antibody	No. of patients with high MET	Follow-up (month)	Survival analysis/HR	Co-founders	Blinding of MET evaluation
Sun	2013	China	2004–2008	61(47/14)	Mean 58.7 range 32–75	NSCLC	I–IV	IHC	Paraffin	> 3 score	Rabbit polyclonal; Santa Cruz, CA	36 (59%)	Mean 29.6±14.7	R (U,M)	TNM stage, MET expression	NA
Dziadziuszko	2012	USA	NA	189(144/45)	Mean 64 range 37–85	NSCLC	I–IV	IHC	Paraffin	Median score 60; range (0–400)	catalog # 7904430, rabbit monoclonal antibody; Tucson, AZ	83 (44%)	Median 5.3 y; range 1.1–6.9 y	R (U)	-	NA
Tsuta	2012	Japan	1997–2007	883(563/320)	Mean:61.7; range:30–88	NSCLC	I–IV	IHC	Paraffin	≥10%	Clone SP44; Ventana	196 (22.2%)	Median 53.3; range 0.3–150	R (U)	-	NA
Tachibana	2012	Japan	2001–2008	106(51/55)	Median 64 range 31–89	ADC	I–III	IHC	Paraffin	≥40% tumor cells	Rabbit polyclonal, Gumma, Japan	30 (28%)	Median 50; range 5–99	R (U)	-	Yes
Park	2012	Korea	Sep.1994– Dem.2001	380(308/72)	Mean 62 range 54–67	NSCLC	I–IV	IHC	Paraffin	4 to 12 score	3D4, rabbit polyclonal, 1:100; San Francisco, CA	52 (13.7%)	Mean 42.2; range 1–167	R (U,M)	Age, sex, smoking, histology, stage, MET IHC, EGFR IHC, and EGFR FISH	Yes
Hu	2012	China	Jan.2003– Jan.2006	103(77/26)	Median: 60	NSCLC	I–III	IHC	Paraffin	≥3 score	Rabbit polyclonal, China	71 (68.9%)	Range: 4–60	R (M)	Differentiation, T stages, Lymphatic metastasis,TNM stages, MACC1	Yes
Onitsuka	2010	Japan	2003–2007	183(102/81)	Mean 68.5 range 23–88	ADC	IA–IIIB	IHC	Paraffin	3 to 8 score	sc-7949, rabbit polyclonal, 1:100; Santa Cruz, CA	104 (57%)	Median 34.2; range 1.0–70.0	R (U,M)	Sex, age, stage, EGFR and KRAS mutations, p-MET, HGF expression	Yes
Liu	2010	China	Mar.2001– Mar.2004	98(62/36)	Median 56 range 25–73	NSCLC	I–IV	IHC	Paraffin	≥25%	Rabbit polyclonal, China	62 (63.3%)	Median 46; range 8–69	R (M)	Stage	Yes
Ruiz	2009	USA	NA	178 (127/51)	NA	NSCLC	I–III	IHC	Paraffin	Score >5	NA	72(40%)	≥5 years	R (M)	Stage, KARS mutation, Type of resection, EGFR.	Yes
Nakamura	2007	Japan	1999–2003	130(82/48)	Mean:65.4; range:36–81	ADC	IA–IIIB	IHC	Paraffin	≥2+	Rabbit polyclonal, Gumma, Japan	47 (36.1%)	Median 31.4; range 0.8–57.3	E (U)	-	Yes
Masuya	2004	Japan	Jan.1993- Mar.2001	88	-	NSCLC	I–IIIB	IHC	Paraffin	Staining intensity≥1 grade	SC-10, rabbit polyclonal, 1:100; Santa Cruz, CA	36 (41%)	Mean: 49.8±36.1	R (U)	-	Yes
Tokunou	2001	Japan	1984–1993	131 (73/58)	Median 59 range 26–80	ADC	I–IV	IHC	Paraffin	More than one microscopic area	Rabbit polyclonal, no. 18321,Gumma, Japan	69 (53%)	Median 5.7 y; range 0.6–12 y	R (U,M)	Stage, nodal involvement, vascular invasion, lymphatic invasion	NA
Takanami	1996	Japan	1982–1989	120 (69/51)	Mean:61; range:28–81	ADC	I–IV	IHC	Paraffin	≥2+	C-28, rabbit polyclonal, 1:50; Santa Cruz, CA	56 (47%)	≥5 years	R (M)	Stage, HGF expression	Yes

NA: not available; NSCLC, non-small cell lung cancer; ADC, adenocarcinoma; IHC, immunohistochemistry; HR: hazard ratio, obtained by estimated (E) or reported in text (R). “M” means the HR come from multivariate analysis, and “U” means HR come from univariate analysis; EGFR, epidermal growth factor receptor; HGF, hepatocyte growth factor.

### Study characteristics

For MET GCN, most studies were retrospective cohorts and only one study was a prospective cohort. A total of 11 studies [11]–[21], analyzing 2,866 patients for MET GCN and OS in patients with NSCLCs. Six of these studies employed FISH [11], [21], [14]–[16], [19], one study employed SISH [18], one study employed BISH [20] and three studies employed RT-PCR [13], [17], [21]. The median study sample size was 189 (range 61–844). Frequencies of high MET GCN ranged from 4% to 22% in the eligible studies. In this analysis, 9 studies (2230 patients, 74%) were conducted in Asian populations [12]–[17], [19], [20], [21], and 2 studies (636 patients, 26%) were conducted in non-Asian subsets [11], [18]. Seven studies involved NSCLCs of all histological subtypes [11],[13],[16]–[20], three studies involved adenocarcinoma [14], [15], [21], two studies involved NSCLCs of all histological subtypes and adenocarcinoma [19], [20], and two studies involved squamous cell carcinoma [12], [19]. A total of 9 studies contained information about all cancer stages (I–IV) [11]–[13], [15]–[20], and 2 studies contained information about cancer stages I–III [14], [21]. A total of 7 of the 12 studies (58.3%) reported that a high MET GCN was a poor prognostic factor for survival [11]–[17], and the remaining 4 studies (41.7%) concluded that no statistically significant effect of a high MET GCN on survival was observed [18]–[21], irrespective of whether these studies used univariate or multivariate analyses. The main features of the eligible studies are summarized in Table 1.

For MET protein expression, all eligible studies were retrospective cohorts. A total of 2,650 patients were included in 13 studies [13], [14], [18]–[20], [21]–[26], [33], [34], with sample sizes ranging from 61 to 883 patients (median 125). In all 13 studies, immunohistochemistry was used to detect MET expression in paraffin-embedded specimens. Eleven studies (2283 patients, 86%) were conducted in Asian populations [13], [14], [19], [20], [21], [23]–[26], [33], [34], and two studies (367 patients, 14%) were conducted in non-Asian subsets [18], [22]. Overall, eight studies involved NSCLCs of all histological subtypes [13], [18]–[20], [22], [23], [26], [33], and five studies involved adenocarcinoma [14], [21], [24], [25], [34]. Seven studies investigated patients at all cancer stages (I–IV) [13], [18]–[20], [24], [25], [26], whereas six studies concerned patients at stages I–III (include IA-IIIB) [14], [21]–[23], [33], [34]. Eight of 13 studies identified high MET protein expression as an indicator of poor prognosis [13], [19], [21]–[26], and the remaining 5 studies showed no statistically significant effect of high MET expression on survival [14], [18], [20], [33], [34], irrespective of whether these studies used univariate or multivariate analyses. The main features of the 13 eligible studies are summarized in Table 2.

### Qualitative assessment

The study quality was assessed using the Newcastle–Ottawa quality assessment scale, generating scores ranging from 4 to 9 (with a mean of 5.85), with a higher value indicating better methodology. The results of quality assessment are shown in supplementary Table S1.

### Impact of MET gene copy number on survival

**Figure 2:**
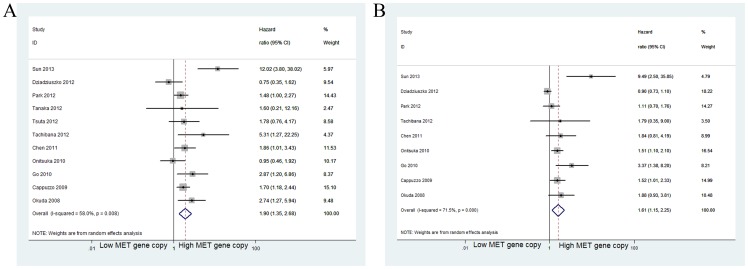
Meta-analysis of effects of the MET gene copy number on overall survival of patients with non-small cell lung cancer (NSCLC). Forest plot showing (A) the combined relative HR for OS by univariate analysis; (B) the combined relative HR for OS by multivariate analysis.

For OS, the estimated pooled HR for increased MET GCN, using univariate and multivariate analyses, was 1.90 (95% CI: 1.35–2.68; *p*<0.001) in eleven studies [11]–[21] and 1.61 (95% CI: 1.15–2.25; *p* = 0.005) in nine studies [11]–[14], [16]–[19], [21]. There was heterogeneity between studies for both univariate (*I^2^* = 58.0%, *p* = 0.008) and multivariate (*I^2^* = 71.5%, *p*<0.001) analyses (Figure 2). Further analysis showed that the observed heterogeneity reflected the inclusion of the studies by Sun et al [13] and Dziadziuszko et al [18] When these studies were excluded from the meta-analysis, less heterogeneity was observed (*I^2^* = 4.2%, *p* = 0.400; *I^2^* = 0%, *p* = 0.488), and the pooled results remained practically unchanged (HR for univariate analysis: 1.74, 95% CI: 1.40–2.15, *p*<0.001; HR for multivariate analysis: 1.53, 95% CI: 1.26–1.87, *p*<0.001).

**Figure 3:**
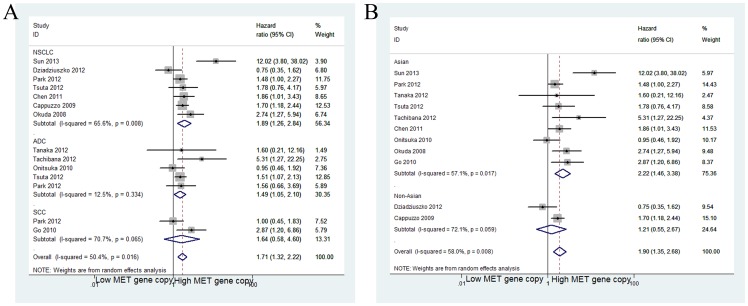
Forest plot (A) assessing MET gene copy number in NSCLC stratified by histological subtypes; Forest plot (B) assessing MET gene copy number in NSCLC stratified by ethnic source.

**Figure 4:**
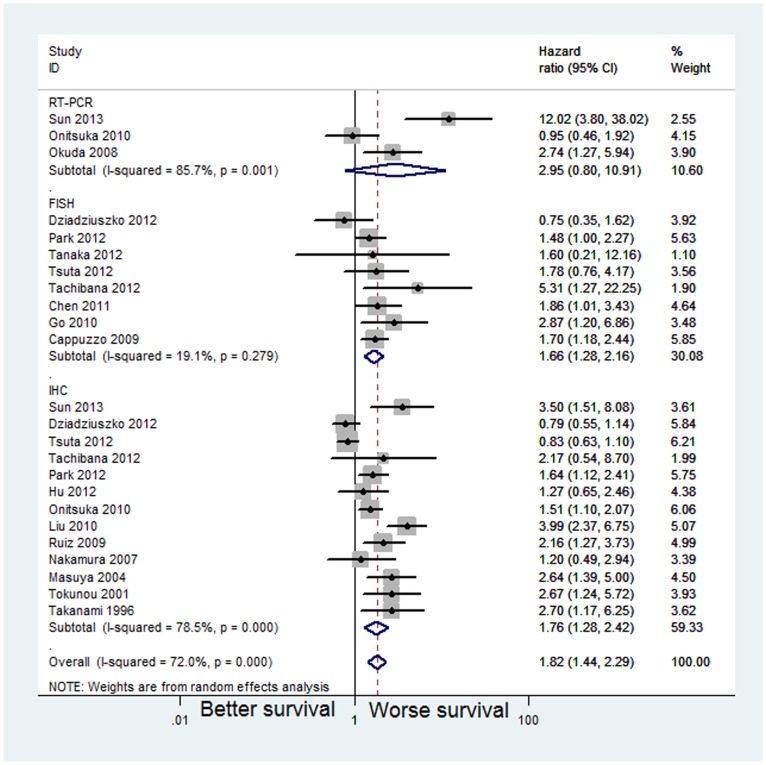
Meta-analysis that according to different methods of MET detection used.

When grouped according to histological subtypes, the combined HR for the NSCLC studies was 1.89 (95% CI: 1.26–2.84), the pooled HR for adenocarcinoma was 1.49 (95% CI: 1.05–2.10) and the combined HR for squamous cell carcinoma was 1.64 (95% CI: 0.54–4.60) (Figure 3) (Table 3). For Asian populations, the increased MET GCN was significantly associated with decreased OS in nine studies (HR = 2.22; 95% CI 1.46–3.38; *p*<0.001), but these results were not observed for non-Asian populations (HR = 1.21; 95% CI 0.55–2.67; *p* = 0.630) (Figure 3) (Table 3). When grouped according to the different methods for determining the MET GCN, the combined HRs for the FISH (including SISH and BISH) and RT-PCR studies were 1.66 (95% CI: 1.28–2.16) and 2.95 (95% CI: 0.80–10.91), respectively (Figure 4).

**Table 3:** Main meta-analysis results.

		Random-effects model		Fixed-effects model		Heterogeneity	
Analysis (overall survival)	*N*	HR (95% CI)	*p*	HR (95% CI)	*p*	*I^2^* (%)	*p*
**MET gene copy number**							
Univariate analysis	11	1.90(1.35–2.68)	<0.001	1.73(1.42–2.11)	<0.001	58%	0.008
All studies except Sun, Dziadziuszko	9	1.74(1.40–2.15)	<0.001	1.73(1.40–2.13)	<0.001	4.2%	0.400
Multivariable analysis	9	1.61(1.15–2.25)	0.005	1.21(1.05–1.40)	0.008	71.50%	<0.001
All studies except Sun, Dziadziuszko	7	1.53(1.26–1.87)	<0.001	1.53(1.26–1.87)	<0.001	0.00%	0.488
**MET protein expression**							
Univariate analysis	9	1.52(1.08–2.15)	0.017	1.25(1.07–1.45)	0.004	75.80%	<0.001
All studies except Tsuta	7	1.84(1.45–2.33)	<0.001	1.78(1.45–2.18)	<0.001	15.20%	0.314
Multivariable analysis	8	2.18(1.60–2.97)	<0.001	1.98(1.64–2.38)	<0.001	57.50%	0.021
All studies except Onitsuka, Liu	6	2.07(1.52–2.81)	<0.001	2.00(1.55–2.57)	<0.001	25.90%	0.24
**Detecting method**							
FISH (include BISH and SISH)	8	1.66(1.28–2.16)	<0.001	1.65(1.33–2.04)	<0.001	19.1%	0.154
RT-PCR	3	2.95(0.80–10.91)	0.106	2.20(1.37–3.55)	0.001	85.7%	0.001
IHC	13	1.76(1.28–2.42)	<0.001	1.42(1.24–1.63)	<0.001	78.5%	<0.001
**Histology**							
MET gene copy number							
NSCLC	7	1.89(1.26–2.84)	0.002	1.73(1.39–2.14)	<0.001	66.60%	0.008
ADC	5	1.49(1.05–2.10)	0.025	1.48(1.12–1.97)	0.006	12.50%	0.334
SCC	2	1.64 (0.58–4.60)	0.35	1.51(0.88–2.61)	0.137	70.70%	0.065
MET protein expression							
NSCLC	8	1.72(1.10–2.69)	0.017	1.33(1.14–1.56)	<0.001	85%	<0.001
ADC	5	1.69(1.31–2.19)	<0.001	1.69(1.31–2.19)	<0.001	0%	0.441
SCC	-	-	-	-	-	-	-
**Country**							
MET gene copy number							
Asian	9	2.22(1.46–3.38)	<0.001	1.90 (1.49–2.43)	<0.001	57.10%	0.017
Non-Asian	2	1.21(0.55–2.67)	0.630	1.33 (0.98–1.81)	0.054	64.20%	0.037
MET protein expression							
Asian	11	1.89(1.34–2.68)	<0.001	1.52(1.31–1.77)	<0.001	76.60%	<0.001
Non-Asian	2	1.28(0.48–3.43)	0.623	1.08(0.80–1.47)	0.603	89.10%	0.002

N: number of studies; HR: hazard ratio; RT-PCR, real-time polymerase chain reaction; FISH, fluorescent in situ hybridization; SISH, silver in situ hybridization; BISH, bright-field in situ hybridization; IHC, immunohistochemistry; NSCLC, non-small cell lung cancer; ADC, adenocarcinoma; SCC, squamous cell carcinoma; EGFR, epidermal growth factor receptor; WT, wild type.

### Impact of MET protein expression on survival

**Figure 5:**
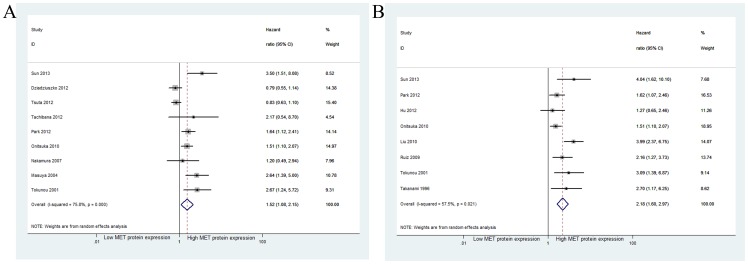
Meta-analysis of effects of the MET protein expression on overall survival of patients with NSCLC. Forest plot showing (A) the combined relative HR for OS by univariate analysis; (B) the combined relative HR for OS by multivariate analysis.

The combined HR for the nine studies [13], [14], [18]–[20], [21], [23], [24], [34] (involving 2151 cases) included in the univariate analysis was 1.52 (95% CI: 1.08–2.15, *p* = 0.0017), indicating that MET overexpression had worse survival impact in patients with NSCLC (Figure 5). Because significant inter-study heterogeneity (*I^2^* = 75.8%, *p*<0.001) was observed, we applied the random-effects model. One study [20] accounted for this heterogeneity; the exclusion of this study from the meta-analysis resulted in less heterogeneity (*I^2^* = 15.2%, *p* = 0.314), and the pooled results remained practically unchanged (HR = 1.84, 95% CI: 1.45–2.33, *p*<0.001).

Eight studies [13], [19], [21], [22], [24], [25], [26], [33] (comprising 1254 cases) were included in the multivariate analysis of MET protein expression for OS. The pooled HR showed a significantly increased risk of mortality in patients with MET positivity (HR = 2.18, 95% CI: 1.60–2.97, *p*<0.00) (Figure 5). Because significant heterogeneity (*I^2^* = 57.5%, *p* = 0.021) was observed among these studies, a random-effects model was applied. The observed heterogeneity might reflect the difference in the populations studied and experimental methods used. Onisuka et al [21] and Liu et al [26] accounted for some of the observed heterogeneity; the exclusion of these studies from the meta-analysis resulted in less heterogeneity (*I^2^* = 25.9%, *p* = 0.24), and the pooled results remained practically unchanged (HR = 2.00, 95% CI: 1.55–2.57, *p*<0.001).

**Figure 6:**
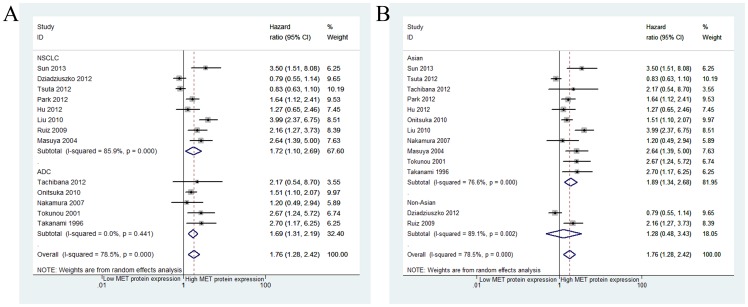
Forest plot (A) assessing MET protein expression in NSCLC stratified by histological subtypes; Forest plot (B) assessing MET protein expression in NSCLC stratified by ethnic source.

When grouped according to histological subtypes, the combined HR for the NSCLC studies was 1.72 (95% CI: 1.10–2.69), and the pooled HR for adenocarcinoma was 1.69 (95% CI: 1.31–2.19) (Figure 6) (Table 3). For Asian populations, MET overexpression was significantly associated with decreased OS in nine studies (HR = 1.89; 95% CI 1.34–2.68; *p*<0.001), but these results were not observed in non-Asian populations (HR = 1.28; 95% CI 0.48–3.43; *p* = 0.623) (Figure 6).

### Sensitivity analyses and publication bias test

The sensitivity analysis indicated that omitting any single study did not influence the pooled HRs. For MET GCN, A more formal evaluation using Egger's test showed no evidence of significant publication bias (*p* = 0.352 for univariate analysis and *p* = 0.063 for multivariate analysis). For the MET protein expression, there was no evidence for significant publication bias (Egger's test: *p* = 0.076 for univariate analysis and *p* = 0.116 for multivariate analysis).

## Discussion

MET has recently received attention as a molecular target for the treatment of NSCLC. Understanding the mechanisms underlying anti-MET therapy requires the correct evaluation of the impact of MET GCN and protein expression on patient survival.

The summary statistics obtained from 18 published studies, including 5,516 patients with NSCLC, showed that high MET GCN or protein expression significantly predicted the poor OS of NSCLC patients (gene copy: HR 1.90, 95% CI 1.35–2.68; protein expression: HR 1.52, 95% CI 1.08–2.15). The subgroup analysis revealed that high MET GCN or protein expression was also significantly associated with poor prognosis in Asian countries (gene copy: HR 2.22, 95% CI 1.46–3.38; protein expression: HR 1.89, 95% CI 1.34–2.68), but the same tendency was not observed in the non-Asian subset (gene copy: HR 1.21, 95% CI 0.55–2.67; protein expression: HR 1.28, 95% CI 0.48–3.43). The present study was performed using univariate analysis, followed by further multivariate analysis. The results of the meta-analysis showed that high MET GCN or protein expression in NSCLC patients was associated with poor OS (univariate analysis). This effect was also significant according to multivariate analysis, showing that the MET GCN or protein expression might be an independent prognostic factor for OS in NSCLC.

The methods used to detect the MET GCN impacted the significance of these results. The combined HRs of 8 FISH (included SISH and BISH) and 3 RT-PCR studies were 1.66 (95% CI: 1.28–2.16) and 2.95 (95% CI: 0.80–10.91), respectively. We observed that FISH, instead of RT-PCR, was the most widely used technology for determining the gene copy number. In clinical practice, although real-time PCR is a simple and quick method, the results do not directly reflect cancer cells because DNA is typically isolated from whole tissue specimens that also contain normal epithelial cells, inflammatory cells, and fibroblasts. FISH is generally accepted as a better technique than RT-PCR for evaluating gene copy number because FISH can be applied to formalin-fixed paraffin-embedded tumor tissues archived for routine pathological diagnosis, thus facilitating the exclusive estimation of tumor cells. Therefore, FISH is the most widely used technique in clinical practice for the detection of gene amplification to determine therapeutic strategies, such as HER2 FISH in breast cancer. The results obtained in the present study showed that increased MET GCN, evaluated using FISH, was a predictor of worse survival in NSCLC. Due to the small number of primary studies using RT-PCR for analysis, the detection of potentially important differences was limited. Moreover, IHC was the method typically used to detect MET protein expression. IHC is the standard method for the evaluation of proteins (e.g., HER2 and EGFR), and there was consistency in the evaluation process among studies. The results of the present meta-analysis showed that MET overexpression was associated with worse survival.

Moreover, the results of the present study demonstrated that high MET GCN or protein expression was an independent negative prognostic factor in NSCLC. However, the prognostic significance of MET GCN according to the histology of NSCLC remains unclear. Go et al [12] reported that SCC patients with MET amplification showed markedly shorter OS than those without MET amplification. In contrast to these results, the systematic review showed that high MET GCN or protein expression is a worse marker of death risk in lung adenocarcinoma than in squamous carcinoma. These results indicated that MET amplification might be involved in the oncogenesis of SCC and ADC. The differences in the two contrasting results were influenced by two SCC studies reporting a correlation between the MET GCN and survival, and these data were not sufficient to determine the prognostic value of MET expression in SCC.

Park et al [19]. demonstrated that MET FISH-positive and MET IHC-positive patients had significantly shorter survival. The results obtained in the present study also provide similar evidence that MET is a negative prognostic factor, further supporting anti-MET strategies, irrespective of MET CGN or MET overexpression. Thus, when patients were divided according to EGFR FISH results, MET positivity had prognostic implications only among EGFR FISH-negative patients. This finding has been consistently reported in recent studies [11], [12], suggesting that anti-MET drugs might be beneficial for EGFR FISH-negative NSCLC patients who are not initially selected for EGFR TKI treatment.

We observed a considerable degree of interstudy heterogeneity. Differences in the detection methods, types and numbers of target genes or antigens, sampling sites and times, and demographic or clinicopathologic data from the included patients, should be considered as potential sources of heterogeneity. In this study, significant heterogeneity was observed among the included studies. Although we used random- and fixed-effects models for pooling data, the source of heterogeneity remained unknown. Moreover, the sensitivity analysis did not clarify the source of the heterogeneity observed in this study. The studies by Sun et al [13] and Dziadziuszko et al [18] primarily accounted for the heterogeneity observed in the MET GCN. Although Sun et al. used RT-PCR, it was not possible to address this technical issue, as these studies used the same primers and other PCR conditions. Dziadziuszko et al [18] used silver *in situ* hybridization (SISH). Silver *in situ* hybridization (SISH) is a new technology for gene copy assessment, with some clinical advantages compared with FISH. First, the samples are analyzed using conventional light microscopy with preserved cell morphology based on automation. The new technology facilitates the evaluation of slides through light microscopy for the simultaneous visualization of amplified signals and cell morphology, overcoming the disadvantage of FISH where the fluorescent signals gradually fade over time. This difference might explain the observed heterogeneity.

Factors associated with immunostaining can also contribute to the observed heterogeneity. Onisuka et al [21] and Liu et al [26] used the same antibodies, but differences in the staining techniques and evaluation criteria for MET positivity might contribute to heterogeneity between studies. The exclusion of this study from the analysis only partially reduced the heterogeneity, potentially reflecting immunohistochemistry techniques (various definitions of threshold positivity, use of the mAb at different concentrations and dissimilar staining protocols) or patient characteristics (type of patients, disease characteristics). These factors might not only contribute to the observed statistical heterogeneity but also the clinical heterogeneity. Clinical heterogeneity might result from the different patients (with different age, tumor size, clinical stage, ethnicity, physical condition, etc.), diverse treatment types, various treatment protocols, different dosages and drug types, etc. Moreover, differences in primary antibodies, IHC staining protocols, evaluation standards, and cut-off values for high MET expression might also contribute to heterogeneity. Thus, additional multicenter studies using standardized methods are encouraged.

Some limitations of this meta-analysis need to be discussed. First, our meta-analysis is based on data from trials whose results have been published, and we did not obtain individual patient data. Use of individual patient data may further enhance the accuracy and reduce the uncertainty of the estimates. Second, significant heterogeneity was observed among the included studies. Factors associated with variability in definitions of end point, measurements, and experimental design may contribute to the heterogeneity. Therefore, validation of the prognostic power of MET GCN or protein expression should be conducted through large multicenter prospective studies based on homogeneous populations. Third, the number of studies concerning MET and the effectiveness of therapy (such as chemotherapy or EGFR TKI treatment) was too small to perform a pooled analysis. In the present study, due to the incompleteness of clinicopathological parameters, we did not perform subgroup analyses between MET GCN and clinicopathological parameters or between protein expression and clinicopathological parameters. Fourth, negative studies are less frequently published or published with less detailed results, making these studies less assessable, potentially leading to some bias.

Despite these limitations, this meta-analysis had some advantages. First, the results obtained from the random-effects model were similar to those obtained from the fixed-effects model, indicating that the statistical results were robust. Second, the results of the sensitivity analysis were not materially altered and did not draw different conclusions, indicating that the initial results were strong. Third, Egger's test did not detect publication bias, indicating that the obtained results were not biased. Moreover, the study quality scores, assessed using the Newcastle–Ottawa quality assessment scale, were >5, suggesting that the results of the present meta-analysis were convincing.

In conclusion, this meta-analysis indicated that increased MET GCN and protein expression was significantly associated with poorer survival in patients with NSCLC; this information could potentially further stratify patients in clinical treatment.

## Supporting Information

Table S1 **Assessment of Newcastle-Ottawa Scale methodological quality of cohort studies.**
(DOC)

Checklist S1 **PRISMA Checklist.**
(DOC)
