# Supplementary material for: Prognostic Value of MET Gene Copy Number and Protein Expression in Patients with Surgically Resected Non-Small Cell Lung Cancer: A Meta-Analysis of Published Literatures
Source: PLoS One. 2014 Jun 12;9(6):e99399. doi: 10.1371/journal.pone.0099399 (PMC4055667; doi:10.1371/journal.pone.0099399)
Supplement: Table S1 — Assessment of Newcastle-Ottawa Scale methodological quality of cohort studies. (DOC) [file pone.0099399.s001.doc]

| Supplementary Table S1 Assessment of Newcastle-Ottawa Scale methodological quality of cohort studies | | | | | | | | | |
| --- | --- | --- | --- | --- | --- | --- | --- | --- | --- |
| **Study ^1^** | **Selection** |  |  |  | **Comparability** | **Outcome** |  |  | **Score ^2^** |
|  | **Representativeness of the exposed cohort** | **Selection of non exposed cohort** | **Ascertainment of exposure** | **Outcome not present at start** |  | **Assessment of outcome** | **Follow-up length** | **Follow-**  **up adequacy** |  |
| Sun | **★** | **★** | **★** | **★** | **★★** | - | **★** | **★** | **8** |
| Dziadziuszko | **★** | **★** | **★** | - | **★★** | - | **★** | - | **6** |
| Park | **★** | **★** | **★** | **★** | **★** | - | **★** | - | **6** |
| Tanaka | **★** | **★** | **★** | **★** | **★★** | - | **★** | - | **7** |
| Tsuta | **★** | **★** | **★** | **★** | **★★** | **★** | **★** | **★** | **9** |
| Tachibana | **★** | **★** | **★** | - | **★** | **★** | **★** | - | **6** |
| Chen | **★** | **★** | **★** | **★** | **★★** | **★** | **★** | - | **8** |
| Onitsuka | **★** | **★** | **★** | **★** | **★** | - | **★** | - | **6** |
| Go | **★** | **★** | **★** | **★** | **★★** | **★** | **★** | - | **8** |
| Cappuzzo | **★** | **★** | **★** | **★** | **★★** | **★** | **★** | **★** | **9** |
| Okuda | **★** | **★** | **★** | **★** | **★** | **★** | **★** | - | **7** |
| Hu | **★** | **★** | **★** | **★** | **★** | **★** | **★** | - | **7** |
| Liu | - | **★** | **★** | **★** | - | - | **★** | **★** | **5** |
| Zucali | - | **★** | **★** | **★** | **★** | - | - | - | **4** |
| Nakamura | **★** | **★** | **★** | **★** | - | - | **★** | - | **5** |
| Masuya | - | **★** | **★** | **★** | - | - | **★** | - | **4** |
| Tokunou | **★** | **★** | **★** | **★** | **★** | - | **★** | - | **6** |
| Takanami | **★** | **★** | **★** | **★** | - | **★** | **★** | - | **6** |
| **^1^ Newcastle-Ottawa Quality Assessment Scale: study can have 1 star ( ★ ) for meeting each criterion, except that comparability (design or analysis) can have a maximum of 2 stars. For comparability in this study: 1 star if controlled for age; 2 stars if also controlled for other important variables such as Age, sex, smoking, histology, stage, EGFR and KRAS mutations, nodal status, etc.** | | | | | | | | | |
| **^2^ Quality evidence score, study met criteria for selection (4 items), comparability (1 star; upgraded a level for 2 stars), and outcome assessment. Downgrading could be due to design or lack of information in report.** | | | | | | | | | |
